# Fidelity of Target Site Duplication and Sequence Preference during Integration of Xenotropic Murine Leukemia Virus-Related Virus

**DOI:** 10.1371/journal.pone.0010255

**Published:** 2010-04-20

**Authors:** Sanggu Kim, Alice Rusmevichientong, Beihua Dong, Roland Remenyi, Robert H. Silverman, Samson A. Chow

**Affiliations:** 1 Biomedical Engineering Interdepartmental Program, University of California Los Angeles, Los Angeles, California, United States of America; 2 Department of Molecular and Medical Pharmacology, Molecular Biology Institute, and University of California Los Angeles AIDS Institute, University of California Los Angeles School of Medicine, Los Angeles, California, United States of America; 3 Department of Cancer Biology, Lerner Research Institute, Cleveland Clinic, Cleveland, Ohio, United States of America; University of Minnesota, United States of America

## Abstract

Xenotropic murine leukemia virus (MLV)-related virus (XMRV) is a new human retrovirus associated with prostate cancer and chronic fatigue syndrome. The causal relationship of XMRV infection to human disease and the mechanism of pathogenicity have not been established. During retrovirus replication, integration of the cDNA copy of the viral RNA genome into the host cell chromosome is an essential step and involves coordinated joining of the two ends of the linear viral DNA into staggered sites on target DNA. Correct integration produces proviruses that are flanked by a short direct repeat, which varies from 4 to 6 bp among the retroviruses but is invariant for each particular retrovirus. Uncoordinated joining of the two viral DNA ends into target DNA can cause insertions, deletions, or other genomic alterations at the integration site. To determine the fidelity of XMRV integration, cells infected with XMRV were clonally expanded and DNA sequences at the viral-host DNA junctions were determined and analyzed. We found that a majority of the provirus ends were correctly processed and flanked by a 4-bp direct repeat of host DNA. A weak consensus sequence was also detected at the XMRV integration sites. We conclude that integration of XMRV DNA involves a coordinated joining of two viral DNA ends that are spaced 4 bp apart on the target DNA and proceeds with high fidelity.

## Introduction

Xenotropic murine leukemia virus (MLV)-related virus (XMRV) is a new human retrovirus having a 8.65 kbp genome and shares up to 95% overall nucleotide sequence identity with other known MLVs [1]. XMRV was first reported to be associated with prostate cancer from patients homozygous for a defective variant of RNase L (R462Q), a regulated endoribonuclease for single-stranded RNA that functions in the antiviral action of interferon (IFN) [1], [2]. The Arg to Gln substitution at amino acid position 462 (R462Q) of RNase L is a common missense variant (35% allelic frequency), resulting in a 3-fold decrease in catalytic activity compared with the wild-type enzyme [3], [4]. Consistent with the observation that the virus is associated with patients having the homozygous mutant *RNASEL* genotype, XMRV replication *in vitro* is sensitive to IFN-β inhibition [5]. The link between XMRV and prostate cancer suggests that inherited defects of RNase L may enhance susceptibility to XMRV, leading to tumorigenesis. However, detection of XMRV has recently been reported in prostate samples independent of the *RNASEL* genotype [6]. XMRV has also been detected in the blood of patients with chronic fatigue syndrome [7]. The causal relationships of XMRV infection to prostate cancer and chronic fatigue syndrome, as well as the mechanism for virus pathogenicity, have yet to be established. Additionally, several studies have failed to detect XMRV in different European cohorts of patients with either prostate cancer [8] or with chronic fatigue syndrome [9], [10], [11], suggesting that either population differences or environmental factors may modulate the incidence of XMRV infections.

Integration of the cDNA copy of the viral RNA genome is essential for retroviruses to establish a productive infection (for reviews, see reference [12]). However, because of its nonspecific nature, retroviral DNA integration is inherently a mutagenic event. Many retroviruses, especially members of the gammaretrovirus genus, can induce tumors as a consequence of integrating their viral genome into the host cell chromosome and activating proto-oncogenes via promoter or enhancer insertion, a mechanism referred to as proviral insertional mutagenesis [13]. XMRV is a member of the gammaretrovirus family, and does not encode host-derived oncogenes [1]. Genome-wide analyses of XMRV integration sites in a human prostate cell line, DU145, and prostate cancer tissues showed that XMRV integration favors gene-dense regions and genomic features frequently associated with structurally open, transcriptional regulatory regions of a chromosome, such as transcription start sites, CpG islands, and DNase hypersensitive sites [14]. The XMRV integration sites in prostate cancer tissues are further associated with cancer breakpoints, common fragile sites, and microRNA genes. However, no common integration site or integration hotspot has been detected within or near known proto-oncogenes and tumor suppressor genes in both acutely infected cells and cancer tissues [14]. Due to the relatively few integration sites (a total of 14) analyzed thus far in prostate cancer tissues, the role of XMRV infection in causing prostate cancer by insertional mutagenesis is still unclear.

**Figure 1:**
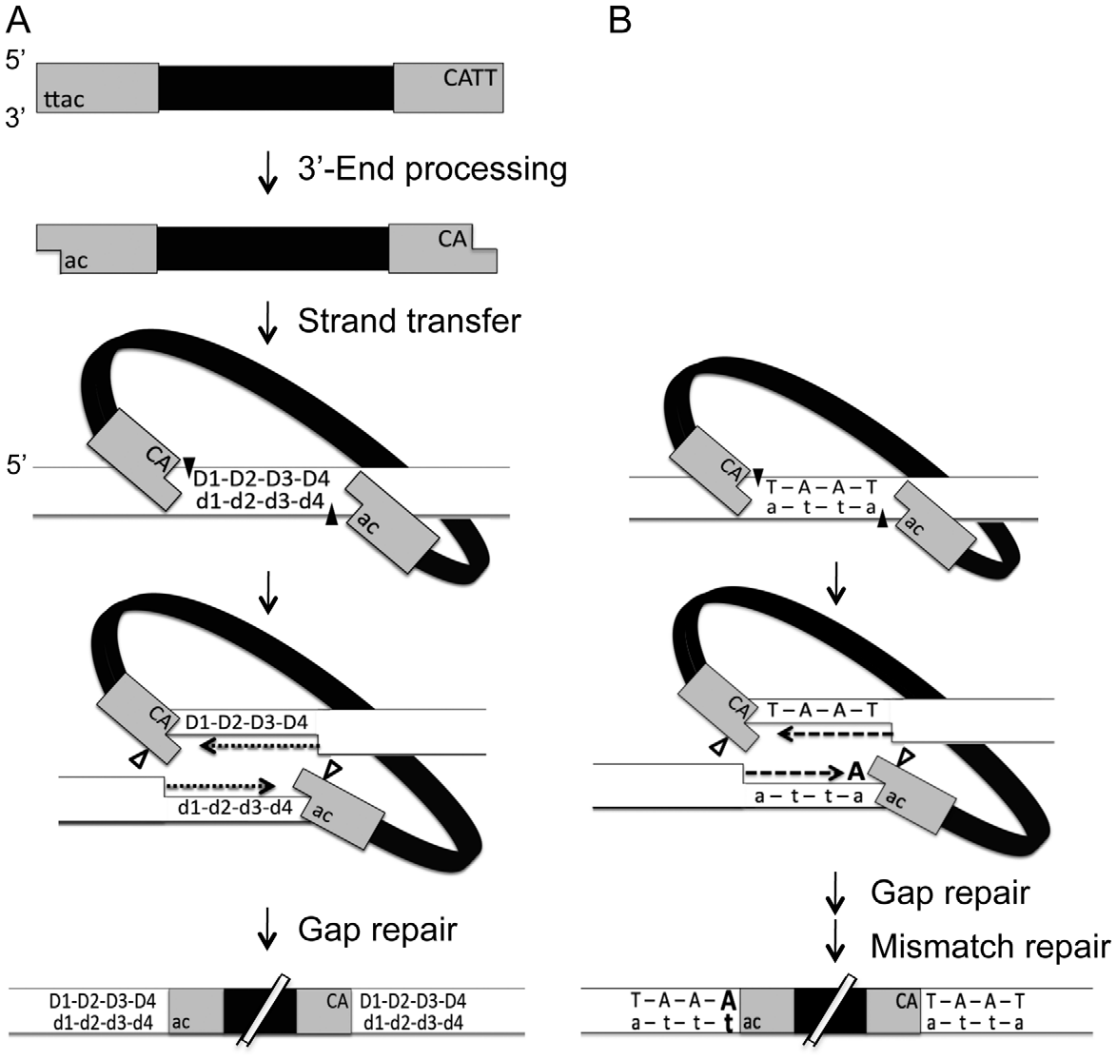
Integration of retroviral DNA and generation of short direct repeats flanking the provirus. (A) DNA breaking and joining steps during integration. Viral and target DNA strands are represented by thick black and parallel lines, respectively, and the viral long terminal repeats (LTRs) are depicted as grey boxes. Nucleotides at the top and bottom strands are denoted by uppercase and lowercase letters, respectively. During 3′-end processing, IN removes two nucleotides from the 3′ end of each strand of linear viral DNA so that the viral 3′ ends terminate with a conserved CA dinucleotide. Closed arrowheads denote the positions of strand transfer, a concerted cleavage-ligation reaction during which IN makes a staggered break in the target DNA. Host DNA repair enzymes fill in the resulting single-stranded gaps, denoted by D1 to D4 in the upper strand and d1 to d4 in the lower strand of target DNA, and remove the two unpaired nucleotides at the 5′ ends of the viral DNA (open arrowheads), thereby generating the short direct repeats flanking the provirus. (B) A potential pathway for generating a base transversion in the short direct repeat during XMRV integration. A coordinated integration of the two viral ends occurred at the 4-bp staggered positions as depicted by the closed arrowheads. During repair of the single-stranded gap adjacent to the upstream LTR, an adenine nucleotide was introduced at the D4 position either by misincorporation or aberrant processing of the unpaired AA-dinucleotide at the viral 5′ end. Subsequent repair of the mismatch resulted in the observed transversion (denoted by bold types).

Integration of retroviral DNA is catalyzed by the viral enzyme integrase (IN) and involves sequential steps of DNA breaking and joining reactions ([12]; and see Fig. 1A). During integrative recombination, the two ends of the linear viral DNA genome are joined in a concerted fashion to staggered sites on the opposite strands of the target DNA. Gap repair of the integration intermediate results in the formation of a provirus that is flanked by short direct repeats of target DNA, a hallmark of retroviral DNA integration [15], [16]. The length of the direct repeats, which varies from 4 to 6 bp among the retroviruses but is invariant for each particular retrovirus, presumably corresponds to the spacing of the staggered target DNA sites that are attacked by IN during integration. Analyses of various proviruses together with the associated flanking DNA sequences have revealed high integration fidelity. For instance, 15 of 15 human immunodeficiency virus type 1 (HIV-1) integration sites [17], [18], [19], 8 of 8 MLV integration sites [20], [21], [22], and 7 of 7 spleen necrosis virus integration sites [23], [24] have the correct length of the target site duplication. However, certain mutations of the viral genome or reaction conditions can lead to uncoordinated integration of the two viral ends and result in deletions, insertions, or other rearrangements of the host DNA [25], [26], [27], [28], [29]. Therefore, in addition to insertional mutagenesis, uncoordinated integration of the two viral ends during integrative recombination may constitute another mechanism that can cause genomic alterations and initiate deleterious events in the infected cell. In this study, we have cloned and determined host DNA sequences flanking XMRV proviruses. We found that integration of XMRV DNA proceeds with high fidelity, and consistently produces a 4-bp direct repeat at the virus-target DNA junctions. Analysis of the 4-bp direct repeats reveals a weak consensus integration sequence.

## Results

### Fidelity and length of target site duplication during XMRV integration

The IN-catalyzed integration of retroviral DNA involves sequential DNA breaking and joining steps (Fig. 1A). To determine the length of the target-site duplication during XMRV integration, we sequenced the stretches of host cell DNA flanking the long terminal repeat (LTR) at each end of a given provirus, and then searched for these flanking sequences within the human genome. To facilitate the analysis, a human prostate cancer cell line DU145 was infected by XMRV and then clonally expanded. Ten infected cell clones were analyzed, and a total of 15 integration site sequences flanking both ends of the XMRV provirus were determined and mapped (Table 1). Three cell clones (C-6, -7, and -8) contained multiple XMRV proviruses, which may have resulted from multiple integration events within the same cell clone or from mixed clonal populations.

**Table 1:** Positions of XMRV integration sites and lengths of the target site sequence duplication.

Cell Clones	Integration Site* (chromosome; nucleotide position)	Duplication Length (bp)
C-1	13; 77,016,416 (+)	4
C-3	2; 33,211,657 (+)	273 †
C-4	5; 34,622,591 (+)	4
C-5	10; 25,254,665 (+)	4
C-6	1; 19,788,033 (+)	4
	2; 19,118,533 (+)	4
C-7	4; 109,005,770 (−)	4
	5; 64,073,721 (+)	4
	9; 94,680,941 (−)	4
	19; 2,119,434 (+)	4
C-8	1; 8,643,694 (+)	4
	1; 9,804,426 (+)	4 ψ
C-9	2; 109,669,551 (−)	4
C-10	6; 30,858,925 (+)	5
C-12	16; 67,648,746 (−)	4

*The nucleotide position corresponds to the position of viral DNA insertion at the top strand of the chromosome indicated. Symbols + and – within the parenthesis indicate the orientation of the viral transcription is the same and opposite, respectively, to the polarity of the top strand. GenBank accession numbers for the integration site sequences are GU816075 to GU816104.

†The left LTR of the provirus contains a 5-bp deletion that includes the conserved CA dinucleotide at the viral end.

ψThe target DNA contains a T to A transversion immediately adjacent to the left LTR (position 4).

Of the 15 XMRV integration sites analyzed, 13 had a 4-bp target site duplication, one site had a 5-bp duplication (clone C-10), and one had a 273-bp duplication (clone C-3). Examination of the viral DNA sequence of the provirus with the 273-bp target site duplication revealed that the left LTR contained a 5-bp deletion at the U3 end that includes a CA dinucleotide that is highly conserved in retroviruses [12]. Deletion or mutation of the CA-dinucleotide in the viral donor DNA substrates significantly reduces the efficiency of coordinated integration of two donor molecules into a target DNA [28], [30], [31], [32], [33]. The U3 end deletion in the left LTR might cause an uncoordinated integration of the two XMRV DNA ends, resulting in staggered breaks that were 273-bp apart. For the 13 proviral integration sites with a 4-bp duplication, 12 had duplication sequences that matched correctly with human genomic DNA sequences. The remaining integration site (from clone C-8) contained a T to A transversion at the position 4 within the direct repeat flanking the left LTR (5′-TAAA), while the direct repeat flanking the right LTR (5′-TAAT) matched correctly with the human genomic DNA (5′-TAAT). Since mismatches in the genome would most likely be repaired by host enzymes before integration, we speculate that the transversion was produced by base misincorporation during gap filling or aberrant processing of the unpaired nucleotides at the viral 5′ end, followed by mismatch repair that fixed the mutation (Fig. 1B).

In addition to the length of the direct repeats, analysis of the 15 integration site sequences showed that all viral sequences, with the exception of the left LTR end of the proviral clone C-3, were terminated with the conserved CA dinucleotide at the 3′ end (data not shown), indicating that the viral DNA ends were correctly cleaved by IN [12]. Based on our analysis that 87% (13 of 15) of the proviruses had a correct 4-bp direct repeat at the integration site, we conclude that the majority of XMRV integration reactions involve a concerted joining of two viral DNA ends that are spaced 4 bp apart on the target DNA.

### Base composition surrounding XMRV integration sites

**Figure 2:**
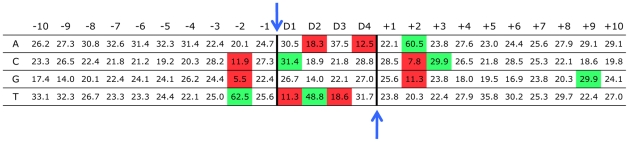
Base composition surrounding XMRV integration sites. Base compositions of the 4-bp target site duplication (positions D1 to D4; demarcated by the thick vertical lines) and 10 bp upstream (positions −1 to −10) and downstream (positions +1 to +10) of the direct repeat were calculated. The datasets include the 13 integration sites with correct 4-bp direct repeat (Table 1), 472 integration sites from acutely infected DU145 cells (GenBank accession numbers EU981292 to EU981799) and 14 integration sites from human prostate cancer tissues (GenBank accession numbers EU981800 to EU981813) [14]. Integration occurs between positions −1 and D1 on the top strand, and between positions D4 and +1 on the bottom strand (blue arrows). Any base in a position that is significantly overrepresented than the random dataset (*P*<0.0001) is highlighted in green, while any base in a position that is significantly underrepresented than the random dataset (*P*<0.0001) is highlighted in red.

Genome-wide analyses of virus-target DNA junctions reveal a weak consensus integration sequence that is nonetheless unique for each retrovirus examined [34], [35], [36], [37], [38]. This consensus integration sequence is generally palindromic. For instance, the consensus integration sequence for HIV-1 and MLV are 5′-GTWAC and 5′-VTAB, respectively (using standard International Union of Biochemistry base codes: B  =  C, G, or T; V  =  A, C, or G; W  =  A or T) [34], [35], [37], [38]. To determine the base composition surrounding the XMRV integration site, the target DNA sequences flanking the proviruses were aligned relative to the integration site (between position −1 and D1; Fig. 2), and the nucleotide frequency of the 4-bp direct repeat (positions D1 to D4; Fig. 2) and the positions 10 bp upstream (positions −1 to −10) and 10 bp downstream (positions +1 to +10) of the direct repeat were calculated. In addition to the 13 integration site sequences from the cell clones, the analysis included a dataset containing 472 XMRV integration sites from acutely infected DU145 cells and 14 integration sites from human prostate cancer tissues [14].

Comparison of the nucleotide frequency at each position to the value of a random dataset generated *in silico* led to identification of a 5′-CTVB consensus sequence (*P*<0.0001). Among all the retroviruses analyzed, the consensus integration site sequence of XMRV is most similar to that of MLV [34], [35], [38]. Both XMRV and MLV generate a 4-bp target site duplication with thymine favored at the D2 position and adenine disfavored at the D4 position. In addition, thymine was disfavored at the D1 position for both XMRV and MLV. At position D3 of the XMRV integration site sequence, although the only statistical significance at *P*<0.0001 was the underrepresentation of thymine, adenine was significantly favored at *P*<0.005. In addition to the 4-bp direct repeat, many positions upstream and downstream of the direct repeat had nucleotide frequencies that were significantly overrepresented (e.g. cytosine and guanine at positions +3 and +9, respectively) or underrepresented (e.g. guanine at position −2) when compared to the random *in silico* control. Furthermore, some of the positions with significantly different representation showed symmetry, such as adenine being favored at position +2 and the corresponding thymine being favored at position −2. Other positions exhibiting a distinct nucleotide preference, however, did not show this symmetry; for example, cytosine was favored at position +3, but guanine was not favored at position −3.

## Discussion

XMRV is a newly discovered gammaretrovirus that has been associated with prostate cancer and chronic fatigue syndrome in humans [1]. An important question is whether XMRV has a causal role in initiation or progression of either of these two diseases. In this study, we investigated if integration of XMRV DNA into the host cell chromosome can cause genetic alterations that may subsequently lead to human disease. During integration, the two ends of the linear viral DNA are joined to staggered sites on the opposite strands of the target DNA [12]. Subsequent strand separation and gap repair lead to the presence of short direct repeats flanking the proviral DNA [15], [16]. Therefore, the length of the direct repeats presumably corresponds to the spacing of the two viral ends on target DNA during integrative recombination catalyzed by IN. Analyses of various proviruses have revealed that the length of target site duplication, though varying from 4 to 6 bp among the different retroviruses examined, is invariant for each particular retrovirus [12], [23], [39]. The high fidelity of the direct repeat length supports the notion that IN multimers form a stable complex with viral and target DNA and catalyze coordinated processing and integration of the two viral DNA ends [30], [33], [40], [41], [42]. In addition, reaction conditions *in vitro* and *in vivo* that promote uncoordinated integration of the two ends often produce deletions and duplications of various lengths in the target DNA [25], [26], [27], [28], [29], [31], [39], [43]. Since the majority of the integrated XMRV contain viral sequences that terminate with the conserved CA dinucleotide and are flanked by a 4-bp direct repeat of target DNA sequence, we conclude that the two viral DNA ends are correctly processed and joined in a coordinated manner to target DNA by IN during XMRV integration.

Although retroviruses can access most of the host genome for integration, selection of particular target sites is not random, and the frequency of use of specific sites varies considerably, with some sites being preferred up to several hundred times greater than random [44], [45], [46]. The mechanism that determines target site specificity is not well understood, and is likely affected by multiple factors [47], [48]. Both *in vitro* and *in vivo* studies have implicated IN as one important determinant in specifying a chromosomal or DNA site for integration. INs of different retroviruses exhibit significant differences in the distribution and preference of integration into an identical target substrate *in vitro* [49], [50], [51], and *in vivo*, a chimeric HIV that encodes IN from MLV integrates preferentially into chromosomal features favored by MLV (i.e. transcription start sites and CpG islands) instead of transcription units as favored by HIV-1 [39]. Although primary DNA sequence is likely not a dominant factor in determining target site specificity, genome-wide analyses of virus-target DNA junctions reveal the presence of weak consensus integration sequences, which are generally palindromic and unique for each retrovirus [34], [35], [36], [37], [38], [52], [53], [54], [55]. A weak palindromic consensus sequence is also detected among the XMRV integration sites. We hypothesize that integration of retroviral DNA into a host DNA site depends on the specific interaction between IN and target DNA sequences, resulting in each retrovirus having its own unique, though weak, consensus sequence. The consensus sequence for each retrovirus may be a result of favorable interactions between the DNA bases and certain amino acid residues of IN, or may reflect the amenability of the sequence in adopting particular DNA structures favorable for IN binding. For instance, a common mechanism for stimulating HIV-1 integration is DNA bending, which creates a widened major groove at the outer curved face that is favorable for integration [49], [56], [57], [58], [59].

The site and fidelity of integration have significant implications for the fate of both the virus and the host cell. Although the present study shows that XMRV integration proceeds with high fidelity, further analysis of additional XMRV integration sites in human tissues would be necessary to clarify whether insertional mutagenesis plays a pathogenic role during XMRV infection. Many viruses from the gammaretrovirus genus of the *Retroviridae* family, such as MLV, feline leukemia virus, and koala retrovirus, are responsible for leukemogenesis and other diseases in their respective host species [60]. Therefore, the recent evidence of authentic infections of humans by XMRV and the association of XMRV infection with prostate cancer and chronic fatigue syndrome [1], [6], [7] are alarming and warrant further investigations to determine the causal relationship and pathogenic mechanisms.

## Materials and Methods

### Host DNA sequences flanking the XMRV provirus

To determine the length and base composition of the target sequence duplication produced by XMRV integration, ten single-clonal (isogenic) populations of XMRV-infected cells were prepared. Plasmid VP62/pcDNA3.1(−) containing the molecular clone of XMRV [5] was transfected with Lipofectamine 2000 (Invitrogen) into DU145 cells. The transfected cells were cultured with complete RPMI 1640 media for 3 weeks, trypsinized, diluted, and plated in 96-well plates so that the calculated number of cells per well on average would be 0.15, 0.45, 1.5, 4.5 and 15. The media from wells with a single colony were assayed for reverse transcriptase (RT) activities after 17 to 24 days. Based on high RT activities, ten clones were chosen for integration site analysis. For each clonal population, the cellular DNA sequence at the right LTR-host DNA junction was determined using the linker ligation-mediated PCR assay as described below. Based on the sequence information of the right LTR-host DNA junction, the left LTR-host DNA junction was amplified by nested PCR using forward primers that anneal to positions upstream of the left LTR-host DNA junction and reverse primers that anneal to sequences downstream and within the left LTR. XMRV613R (5′-GATCGCCGGCCGGCTTA), which is complementary to nt positions 597 to 613 of XMRV, and XMRV165R (5′-CCTGACTACAGATATCCTGTTT), which is complementary to nt positions 143 to 165, were used as reverse primers for the first and second PCRs, respectively. The PCR product was electrophoresed on a 1.5% agarose gel, and the expected size of DNA band was excised from the gel and extracted using a gel extraction kit (Qiagen). Extracted DNA was cloned into a pCR-Blunt vector using a Zero Blunt PCR Cloning Kit (Invitrogen).

### Linker ligation-mediated PCR assay for cloning XMRV integration sites

The genomic DNA from XMRV-infected cells was isolated with a QIAamp DNA Mini Kit (Qiagen) following the manufacturer's instruction. The assay for determining XMRV integration sites in DU145 cells was performed as described previously [14]. Briefly, genomic DNA from XMRV-infected DU145 cells was digested with *Pst* I, which cuts once in the XMRV genome at nucleotide (nt) position 7,534 and produces on average 4-kbp DNA fragments. After digestion, DNA was denatured and annealed with a biotinylated primer, bXMRV7938 (5′-biotin-ATCCTACTCTTCGGACCCTGT), which is complementary to nt positions 7,938 to 7,958 within the *env* gene (about 160 bp upstream of the right LTR). The annealed primer was extended using the PicoMaxx High Fidelity PCR system (Stratagene) to produce biotinylated double-stranded DNA containing the viral-human DNA junction region. The biotinylated DNA product was then isolated by binding to streptavidin-agarose Dynabeads (Dynal), and digested with *Taq^α^*I (5′-T↓CGA), a 4-bp cutter that does not cleave the viral DNA portion of the biotinylated DNA. Digestion of the human genomic DNA with *Taq^α^*I produces on average 1.9-kbp DNA fragments [61]. After digestion, the integration site-containing DNA was ligated with TaqLinker, which was prepared by annealing BHLinkA (5′-**CG**GATCCCGCATCATATCTCCAGGTGTGACAGTTT) with TaqLinkS (5′-CACCTGGAGATATGATGCGGGATC). The TaqLinker contains a 2-nt 5′-overhang (in bold type) complementary to the *Taq^α^*I -digested biotinylated DNA. The linker-ligated DNA product was amplified by a two-step PCR process. The first PCR was carried out using primers XMRV8415F (5′-AACCAATCAGCTCGCTTCTC) and Linker1 (5′-TAACTGTCACACCTGGAGATA) in a final volume of 300 µl with 0.5 µM of each primer, 0.2 mM of dNTPs, and 12 U *Pfu* DNA polymerase (Stratagene) under the following condition: 2 min of preincubation at 94°C, followed by 29 cycles at 94°C for 30 s, 58°C for 30 s, and 72°C for 4 min. The PCR product was purified using a PCR Purification Kit (Qiagen), and was used as the template for the second PCR with two nested primers, XMRV8535F (5′-CGGGTACCCGTGTTCCCAATA) and Linker2 (5′-TAGATATGATGCGGGATCCG), which anneal downstream of XMRV8415F and Linker1 binding sites, respectively. The condition for the second PCR was identical to the first PCR except being conducted with only 18 cycles. The second PCR product was electrophoresed on a 1.5% agarose gel and DNA bands between 200 bp to 2 kbp were extracted and cloned into a pCR-Blunt vector using a Zero Blunt PCR Cloning Kit (Invitrogen).

### Integration site sequence determination and data analysis

The sequence of the cloned DNA was determined by dideoxy sequencing, and sequencing ambiguities were resolved by repeated sequencing on both strands. The authenticity of the integration site sequence were verified by the following criteria: (i) the sequence contained both XMRV LTR and linker sequence, (ii) a match to the human genome begining after the end of the LTR (5′-…CA-3′) and ending with the linker sequence, and (iii) the host DNA region (containing 20 or more nucleotides) from the putative integration site sequence showed 96% or greater identity to the human genomic sequence. The authenticated integration site sequences were then mapped to the human genome hg18 [University of California, Santa Cruz (UCSC) March 2006 freeze; National Center for Biotechnology Information (NCBI) Build 36.1] using BLASTN program (http://www.ensembl.org) or BLAT (UCSC; http://genome.ucsc.edu).

To determine nucleotide preference at integration sites, the target DNA sequences flanking the viral-host DNA junctions were aligned relative to the point of viral DNA integration. The XMRV integration site datasets used to determine nucleotide preference include the 13 correct integration sites listed in Table 1 (GenBank accession numbers GU816075, GU816076, GU816079 to GU816100, GU816103, GU816104), 472 integration sites from acutely infected DU145 cells (GenBank accession numbers EU981292 to EU981799) [14], and 14 integration sites from human prostate cancer tissues (GenBank accession numbers EU981800 to EU981813) [14]. The nucleotide frequency at each position was calculated and compared to values obtained from a set of 10,000 random positions generated *in silico* by choosing a random number between 1 and 3,093,120,360, which represents the total length of the 22 autosomal chromosomes plus the X-sex chromosome of the human genome. The nucleotide frequencies of the random dataset are 29.8%, 20.4%, 20.5%, and 29.3% for A, C, G, and T, respectively. Statistical difference of nucleotide frequency between XMRV integration site sequences and the random dataset was analyzed at each position using a chi-square test at *P*<0.0001.

### Nucleotide sequences accession numbers

The GenBank accession numbers for integration site sequences from the ten XMRV-infected cell clones listed in Table 1 are GU816075 to GU816104.
